# Supplementary material for: Interpretable and Predictive Models Based on High‐Dimensional Data in Ecology and Evolution
Source: Mol Ecol Resour. 2026 Aug 17;26(6):e70191. doi: 10.1111/1755-0998.70191 (PMC13480524; doi:10.1111/1755-0998.70191)
Supplement: Supplementary file 1 [file MEN-26-e70191-s001.pdf]

Supporting Information for

Interpretable and predictive models based on high-dimensional data in ecology and evolution

List of Tables

S1 Simulation attributes 2

List of Figures

S1 Simulation reducible error 3
S2 F_1 versus out-of-sample R^2 4
S3 Posterior inclusion probability (PIP) thresholds versus F_1 5

Scenario	P	N	$\boldsymbol{\beta}_{\text{causal}}$
Core 1	100	50	0.1
Core 2	100	50	0.3
Core 3	100	50	0.8
Core 4	100	150	0.1
Core 5	100	150	0.3
Core 6	100	150	0.8
Core 7	100	500	0.1
Core 8	100	500	0.3
Core 9	100	500	0.8
Core 10	1,000	50	0.1
Core 11	1,000	50	0.3
Core 12	1,000	50	0.8
Core 13	1,000	150	0.1
Core 14	1,000	150	0.3
Core 15	1,000	150	0.8
Core 16	1,000	500	0.1
Core 17	1,000	500	0.3
Core 18	1,000	500	0.8
Core 19	10,000	50	0.1
Core 20	10,000	50	0.3
Core 21	10,000	50	0.8
Core 22	10,000	150	0.1
Core 23	10,000	150	0.3
Core 24	10,000	150	0.8
Core 25	10,000	500	0.1
Core 26	10,000	500	0.3
Core 27	10,000	500	0.8
Core 28	100,000	50	0.1
Core 29	100,000	50	0.3
Core 30	100,000	50	0.8
Core 31	100,000	150	0.1
Core 32	100,000	150	0.3
Core 33	100,000	150	0.8
Core 34	100,000	500	0.1
Core 35	100,000	500	0.3
Core 36	100,000	500	0.8
Large N 1	1,000	1,000	0.3
Large N 2	1,000	10,000	0.3

Table S1: Specifications for the 38 simulation scenarios, including the number of predictors (P), the number of observations (N), and the effect size of causal predictors ($\boldsymbol{\beta}_{\text{causal}}$). One hundred replicates were used for each scenario.

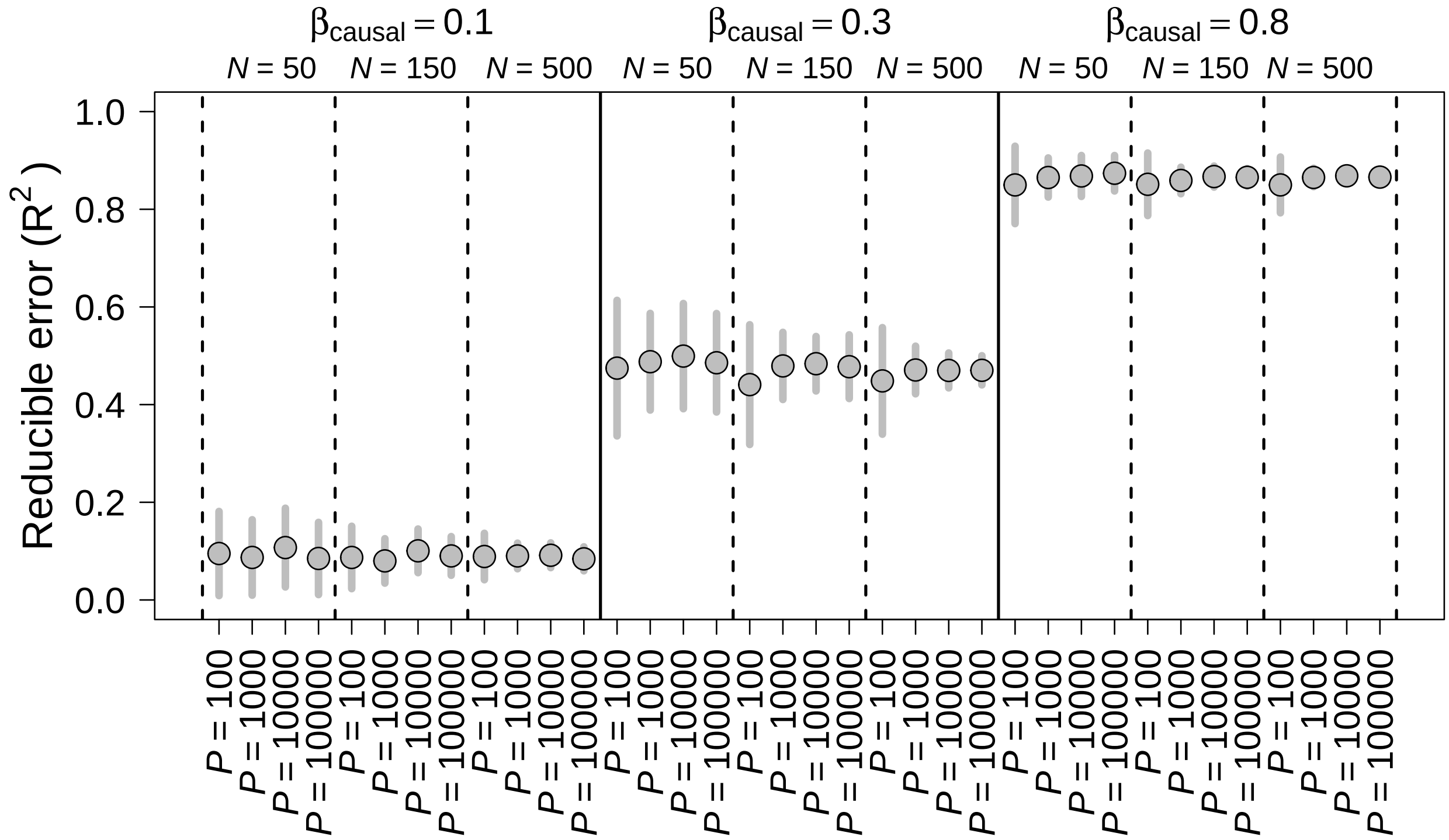

Figure S1: For each core scenario, the reducible error was quantified based on the ability to predict the response using only the 10 causal predictors. Scenarios differed in the number of observations ($N = 50$, 150, or 500), the number of predictors ($P = 100$, 1000, 10000, or 100000), and the effect size of causal predictors ($\boldsymbol{\beta}_{\text{causal}} = 0.1$, 0.3, or 0.8). Median R^2 ($\pm$1sd) from 100 replicates is shown for each scenario.

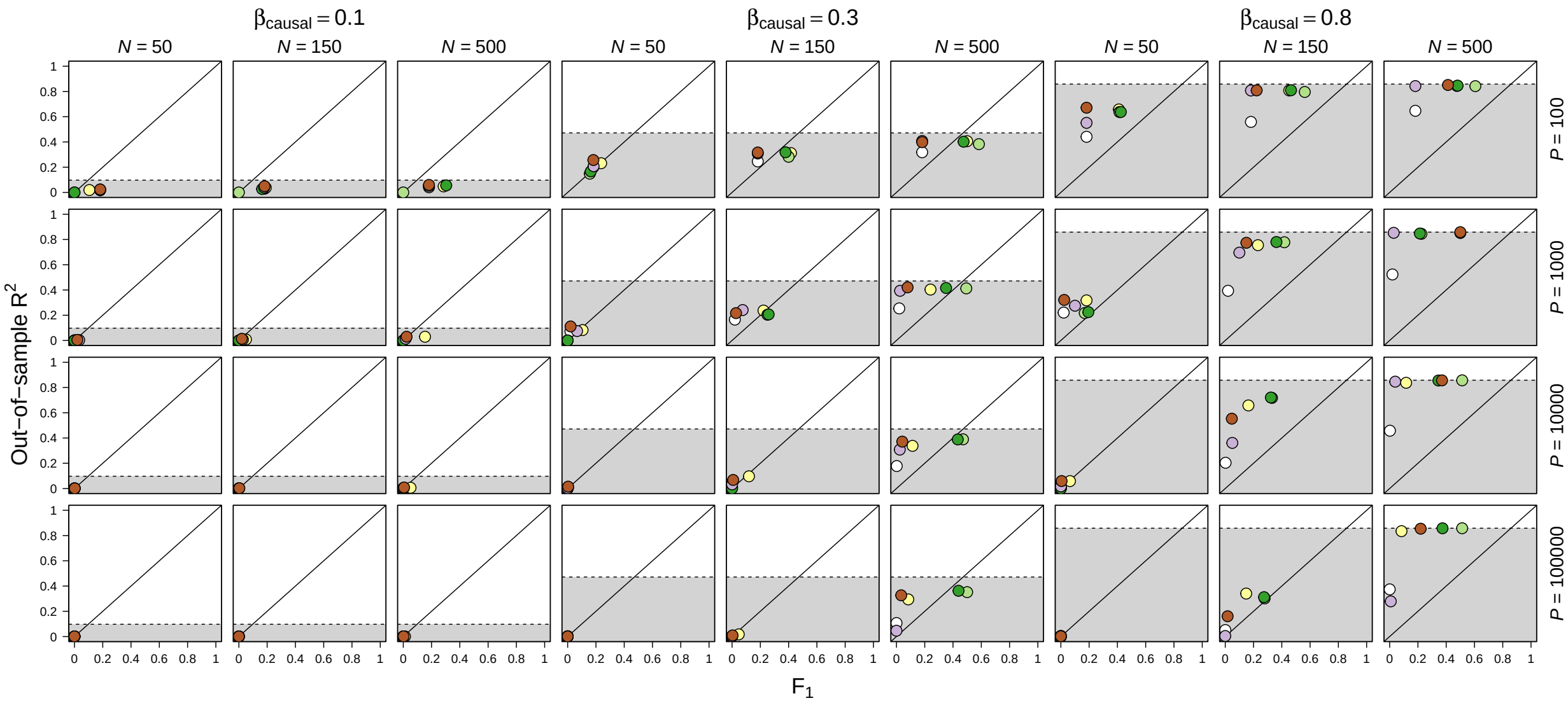

Figure S2: For some scenarios, out-of-sample R^2 converged on the target reducible error R^2 (shown in gray) even when F_1 was relatively low. F_1 is the harmonic mean of precision (i.e., the fraction of selected predictors that are truly causal) and sensitivity (i.e., true positive rate): $\frac{2 \times Precision \times Sensitivity}{Precision + Sensitivity}$. F_1 was only calculated for analyses that can return truly sparse parameter estimates (i.e., $\boldsymbol{\beta} = 0$; BSLMM, Elastic Net, LASSO, Spike-and-slab) or importance values (Random Forest). Each circle represents the median value from 100 replicate simulations. See Fig. 2 in the main text for color legend.

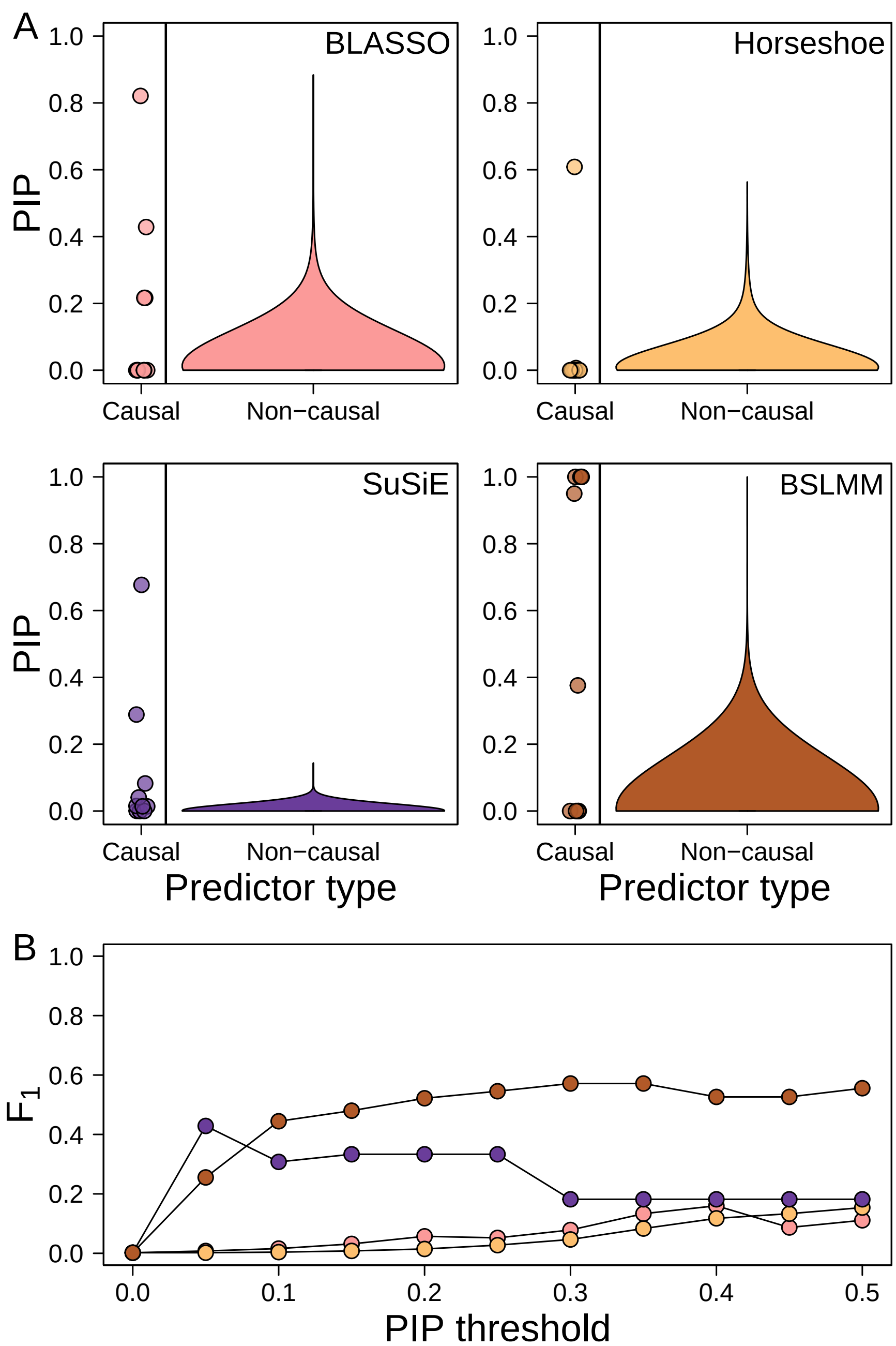

Figure S3: For four Bayesian analyses (BLASSO, Horseshoe, SuSiE, BSLMM), variable selection was also assessed using posterior inclusion probabilities (PIPs) instead of parameter estimates. (A) PIP distributions are shown for the first replicate of scenario 24 (the same example replicate from Fig. 1 in the main text), which had 10 causal predictors ($\boldsymbol{\beta} = 0.8$), 9,990 non-causal predictors ($\boldsymbol{\beta} = 0$), and 150 observations (N). (B) Variable selection was evaluated using F_1, which is the harmonic mean of precision (i.e., the fraction of selected predictors that are truly causal) and sensitivity (i.e., true positive rate): $\frac{2 \times Precision \times Sensitivity}{Precision + Sensitivity}$. The use of a small PIP threshold of 0.05 (i.e., only predictors with PIP > 0.05 are scored as positives) improved variable selection for BSLMM and SuSiE, whereas larger thresholds were needed to recover more limited gains for BLASSO and Horseshoe.
